# Environmentally Associated Developmental Reprogramming from Mycelial Growth to Fruiting Body Formation in *Morchella eximia* Under Off-Season Industrial Cultivation

**DOI:** 10.3390/jof12090665

**Published:** 2026-09-03

**Authors:** Mengjie Gong, Jiayao Lin, Jiling Song, Jia Lu, Na Lu, Jing Yan, Ya Xin

**Affiliations:** 1 Hangzhou Academy of Agricultural Sciences, Hangzhou 310024, China; mjgong@zju.edu.cn (M.G.); ljymumu0215@126.com (J.L.); songjiling860605@163.com (J.S.); lujia920316@163.com (J.L.); 13738068366@163.com (N.L.); yj2006@hz.cn (J.Y.); 2 Engineering Research Center of Ministry of Education of China for Food and Medicine, Jilin Agricultural University, Changchun 130118, China

**Keywords:** *Morchella eximia*, G10, industrial cultivation, transcriptome, metabolome, reproductive development

## Abstract

Morels (*Morchella* spp.) are highly valued edible fungi, but their cultivation remains largely dependent on seasonal field conditions. In this study, we established a stable off-season industrial cultivation system for *Morchella eximia* G10 through precise environmental regulation, including substrate and ambient temperature, humidity, and irrigation management. Based on this controllable cultivation platform, integrated transcriptomic and metabolomic analyses were conducted to explore developmental reprogramming from vegetative growth to fruiting body maturation. Metabolomic profiling was performed only at the reproductive stages, whereas transcriptomic analysis covered both vegetative and reproductive stages. Low-temperature stimulation was associated with marked increases in the expression of genes involved in cell wall remodeling, carbohydrate metabolism and stress responses, together with downregulation of genes annotated with oxidoreductase- and hydrolase-related functions. The transition to the primordium stage represented the most dynamic developmental transition and was associated with transcriptional regulation, cytoskeletal organization, and signal transduction pathways. Subsequently, distinct developmental trajectories were observed between pileus and stipe tissues, suggesting tissue-specific regulatory programs during fruiting body maturation. In the pileus, early amino acid- and nitrogen-related metabolism was coordinated with transcriptional changes, followed by a shift toward carbon and lipid metabolism. In the stipe, early development featured elevated aminoacyl-tRNA biosynthesis and translational capacity, followed by later adjustments in carbon, sulfur and lipid metabolism. These results provide a tissue-resolved multi-omics view of developmental reprogramming during the cold-treatment-associated transition from vegetative growth to fruiting in *Morchella* under controlled off-season cultivation. By linking environmental cues with stage- and tissue-specific transcriptional and metabolic programs, this work provides additional insights into reproductive development and extends previous descriptive omics studies, while offering a molecular basis for optimizing environmental regulation and improving the stability of industrial morel production.

## 1. Introduction

Morels (*Morchella* spp.) are highly prized edible and medicinal fungi due to their unique flavor, rich nutritional composition, and diverse bioactive compounds [1,2,3,4]. In recent years, successful artificial cultivation has facilitated the transition of morels from a wild-harvested delicacy to an economically important cultivated crop. Currently, morel cultivation relies primarily on season-dependent field-based systems using simple plastic tunnels, with commercial production dominated by a few cultivated species, including *Morchella sextelata*, *Morchella eximia*, and *Morchella importuna* [5,6,7,8,9]. However, these production systems remain highly dependent on environmental conditions, particularly temperature, making yields vulnerable to unfavorable weather and seasonal constraints [10,11]. Developing off-season industrial cultivation systems that allow precise environmental regulation is therefore an important step toward more stable and predictable morel production.

Off-season industrial cultivation has only recently been explored in morels and aims to overcome seasonal limitations through precise regulation of environmental parameters. Successful year-round production of other commercially cultivated edible mushrooms, including *Stropharia rugosoannulata* and *Pleurotus* spp., has demonstrated the feasibility of using controlled temperature, humidity, ventilation, and light to regulate fungal growth and fruiting [12,13,14,15,16]. These systems provide a useful framework for developing controllable production strategies for morels. To address this challenge, we established an off-season industrial cultivation system for *Morchella eximia* in Hangzhou, China, by regulating key environmental parameters, including substrate and air temperature, humidity, and irrigation. The resulting cultivation platform enabled fruiting under off-season conditions and provided a relatively stable environmental background for examining developmental molecular changes with reduced variation arising from uncontrolled seasonal conditions.

The development of morel fruiting bodies is a complex and dynamic process involving coordinated cellular differentiation, tissue specialization, and metabolic reprogramming. Morphologically, it spans multiple distinct stages, including vegetative mycelial growth, primordium initiation, and progressive differentiation into mature fruiting bodies with distinct pileus and stipe structures [17,18]. The pileus and stipe perform distinct biological functions during fruiting body development [17,19,20,21]. The pileus serves as the reproductive structure responsible for ascospore formation and quality traits, whereas the stipe primarily contributes to structural support and nutrient transport [22,23]. These functional differences suggest that pileus and stipe differentiation may involve distinct developmental and metabolic programs.

Previous studies have identified several pathways potentially involved in fruiting body development. For instance, comparative transcriptomic analysis between mycelial and young fruiting body stages of *M. importuna* suggested that genes related to carbohydrate catabolism, energy metabolism, and oxidoreductase activity are upregulated during early fruiting [24]. Similarly, integrated transcriptomic and proteomic analyses in *M. sextelata* revealed enrichment in glycometabolism pathways, including starch and sucrose metabolism [25]. Another multi-omics study across mycelial, primordium, young fruiting body, and mature fruiting body stages of *M. sextelata* further highlighted carbohydrate, amino acid, and lipid metabolism as key processes, with the primordium stage being the most transcriptionally and metabolically dynamic [26]. Together, these studies establish that transcriptomic and metabolomic approaches can provide complementary information on the molecular changes associated with Morchella development.

Despite recent advances in omics-based studies of *Morchella*, several gaps remain in our understanding of the molecular regulation underlying fruiting body development. Most published transcriptomic and metabolomic investigations have been conducted under traditional field cultivation conditions or in wild populations, where environmental variation may affect developmental and molecular responses [24,25,26,27]. Moreover, these studies have generally covered only limited developmental stages [25,27], and mycelial samples were often obtained from Petri dish cultures rather than practical cultivation substrates [24,26,28]. Fruiting bodies have also typically been analyzed as whole tissues without separating pileus and stipe samples [25,26], potentially obscuring tissue-specific developmental programs. However, the pileus and stipe represent two functionally distinct developmental compartments with different biological roles in reproduction, structural support, and nutrient allocation. Their differentiation therefore likely involves distinct transcriptional and metabolic programs rather than simply reflecting differences in tissue morphology. Thus, the available datasets have generally not addressed the distinct developmental trajectories of different fruiting body tissues, particularly those of the pileus and stipe, while the relatively limited number of developmental stages or sampling time points in several previous studies restricts the resolution of dynamic molecular changes during tissue differentiation and maturation.

Low-temperature treatment represents an important environmental cue for inducing reproductive development in many edible fungi. However, its role in initiating fruiting under controlled industrial cultivation conditions has not been systematically investigated in *Morchella*. In *Pleurotus tuoliensis* and *Flammulina velutipes*, cold-induced fruiting has been reported to be associated with convergent transcriptional responses involving calcium signaling, MAPK pathways, and cell wall and membrane processes, together with changes in carbohydrate and protein metabolism and molecular chaperones [29,30,31]. These findings suggest that low temperature may serve as an important environmental cue linking external temperature changes to reproductive development, but whether similar regulatory responses operate during cold-induced fruiting in *Morchella* remains unknown.

To address these knowledge gaps, we systematically profiled *Morchella eximia* across six developmental stages, spanning vegetative mycelial growth to mature fruiting bodies, under a controlled off-season industrial cultivation system. Pileus and stipe tissues were separately collected after tissue differentiation, and integrated transcriptomic and untargeted metabolomic analyses were performed to characterize developmental and tissue-specific molecular and metabolic reprogramming. Together, the study provides a developmental and tissue-resolved molecular framework for understanding *M. eximia* fruiting under controlled off-season industrial cultivation conditions, while extending previous multi-omics investigations of *Morchella*.

## 2. Materials and Methods

### 2.1. Fungal Strain and Cultivation Conditions

*Morchella eximia* (strain G10) was used in this study. Cultivation was carried out in Hangzhou, China (119.367° E, 29.383° N), under a controlled industrial system. Mycelia were cultivated on a mixed substrate consisting of peat soil:perlite:garden soil (1:1:1, *v*/*v*/*v*). During the mycelial growth stage, the air temperature was maintained at approximately 13–15 °C, relative humidity at 80–95%, and CO_2_ concentration at 500–1000 ppm under dark conditions. At 52 days after sowing (DAS), the cultivation temperature was initiated by reducing the cultivation temperature to approximately 6 °C and maintaining it for 2–5 days, depending on the developmental status of the cultures, to induce the transition from vegetative to reproductive development. In the present cultivation cycle, the minimum recorded air and substrate temperatures during this period were 3.24 °C and 6.76 °C, respectively, with the slower decrease in substrate temperature reflecting its thermal buffering capacity. After cold treatment and heavy irrigation, fruiting management was conducted at 13–15 °C, 85–95% relative humidity, and 500–1000 ppm CO_2_, with light provided at 800–1500 Lux for 10–14 h per day. Off-season fruiting was successfully achieved during the summer–autumn period (September to November, 2025). Due to the high ambient temperatures in the off-season, actual monitored values may have shown some fluctuations; however, the environmental control systems were set to the target parameters described above.

### 2.2. Sample Collection and Experimental Design

Samples were collected from G10 at six representative developmental stages, including pre-cold-treated mycelia (M1), post-cold-treated mycelia (M2), primordia (F1), early differentiation stage of fruiting bodies (F2), young fruiting bodies (F3), and mature fruiting bodies (F4) (Supplementary Table S1). Specifically, M1 was sampled at 50 DAS, and M2 at 54 DAS. Due to asynchronous fruiting body development under industrial cultivation conditions, primordia, early differentiated fruiting bodies, young fruiting bodies, and mature fruiting bodies coexisted within the cultivation system at the same sampling date. Therefore, these developmental stages were collected simultaneously at 63 DAS and classified according to their morphological characteristics. F1 samples consisted of undifferentiated primordia in which pileus and stipe structures were not yet distinguishable. For the fruiting body stages (F2, F3 and F4), pileus and stipe tissues were carefully separated and collected independently to investigate tissue-specific regulatory patterns. All samples were immediately frozen in liquid nitrogen after harvesting and stored at –80 °C until further analysis.

For each developmental stage or tissue type, fruiting bodies with comparable developmental characteristics were selected from cultivation areas maintained under relatively uniform environmental conditions, including temperature, humidity, light, and ventilation. For each biological replicate, tissues from three to four developmentally comparable fruiting bodies were collected and pooled to reduce variation among individual fruiting bodies. Each pooled sample was treated as one independent biological replicate.

Transcriptomic analysis was performed across all nine developmental sample groups. Due to potential interference from substrate-derived contaminants, metabolomic analysis was conducted only on reproductive stages (F1 to F4), excluding mycelial samples. For transcriptomic analysis, three independently collected pooled biological replicates were prepared for each developmental stage or tissue type, resulting in a total of 27 RNA-seq libraries. For metabolomic analysis, six independently collected pooled biological replicates were prepared for each reproductive sample group, resulting in a total of 42 metabolomic samples.

### 2.3. RNA Extraction and Sequencing

Total RNA was isolated using the SteadyPure RNA Extraction Kit without Lysis Buffer (Accurate Biology, AG21024, Hunan, China) according to the manufacturer’s instructions. A total of twenty-seven poly(A)-enriched RNA sequencing libraries were constructed and subsequently sequenced on an Illumina platform by Novogene Co., Ltd. (Beijing, China). Raw sequencing reads were processed to remove adapter contamination and low-quality sequences, resulting in approximately 6 GB of high-quality paired-end 150 bp clean reads for each biological replicate (Supplementary Table S2). The processed sequencing datasets were used for downstream transcriptomic analyses, and the corresponding raw sequencing data are publicly available in the NCBI Gene Expression Omnibus (GEO) database under accession number GSE333452.

### 2.4. Transcriptome Data Analysis

The filtered reads were aligned to the reference genome *M. eximia* NRRL26621 (JGI assembly Morexi1 v1.0) using HISAT2 v2.2.1 [32], and gene-level read counts were generated with featureCounts [33] with the parameters “-p -t exon-g gene_id”, where paired-end reads were assigned to annotated exons and summarized at the gene level according to the gene_id attribute in the reference GTF file. All other parameters were used as defaults. Downstream analyses were performed in R (v4.3.3) within RStudio (2024.04.2+764). Differentially expressed genes (DEGs) between sample groups were identified using DESeq2 v1.42.0 [34]. DEGs were defined using thresholds of |log_2_(fold change)| ≥ 1 and adjusted *p*-value ≤ 0.05. Transcripts Per Million (TPM) was used for normalizing gene expression level. Principal component analysis (PCA) with three biological replicates were performed using fast.prcomp module from R package gmodels v2.19.1 (https://cran.r-project.org/web/packages/gmodels/index.html, accessed on 1 June 2026), using TPM values of each gene as input. Gene ontology (GO) enrichment analysis of the DEGs was carried out using R package clusterProfiler v4.10.0 [35]. All GO-annotated proteins were used as the background set. Stage-dependent expression pattern clustering was further performed to explore the dynamic transcriptional profiles across developmental stages. Normalized TPM were used as input for clustering analysis using the R package ClusterGVis v0.1.1 [36]. Gene expression trends were grouped into distinct clusters using the k-means algorithm. The optimal number of clusters was determined by the elbow method based on the within-group sum of squares (WSS). The resulting clusters were visualized as expression trend plots, heatmaps, and annotated with corresponding GO enrichment profiles to facilitate functional interpretation of stage-specific transcriptional programs during development.

### 2.5. Real-Time Quantitative PCR Validation

To validate the reliability of the RNA-seq results, representative differentially expressed genes were selected for real-time quantitative PCR (RT-qPCR) analysis. Total RNA (1 μg) from each sample was treated with gDNA Remover and reverse-transcribed into first-strand cDNA using the Accurate Biology Evo M-MLV kit (AG11728, Hunan, China), following the manufacturer’s instructions. RT-qPCR was performed using 2× SYBR Green Pro Taq HS Premi (AG11701, Accurate Biology, Hunan, China) on a real-time PCR system. The amplification program consisted of an initial denaturation at 95 °C for 30 s, followed by 40 cycles of 95 °C for 5 s and 60 °C for 30 s. CYC3 was used as the internal reference gene [37]. Gene-specific primer sequences are listed in Supplementary Table S14. The relative expression levels of target genes were calculated using the 2^−ΔCt^ method, where ΔCt was calculated as the difference between the Ct value of the target gene and that of the reference gene. Relative expression values were subsequently calculated as 2^−ΔCt^ and used for statistical comparison and visualization.

Statistical significance between two groups was determined using a two-tailed Student’s *t*-test, whereas comparisons among multiple developmental stages were performed using one-way analysis of variance (ANOVA). Differences were considered statistically significant at *p* < 0.05.

### 2.6. Metabolite Extraction and UPLC-MS/MS Analysis

Approximately 100 mg of each fruiting body sample was ground in liquid nitrogen and extracted with pre-chilled 80% methanol. Samples were vortexed thoroughly, incubated on ice for 5 min, and centrifuged at 15,000× *g* for 20 min at 4 °C. The supernatants were diluted with LC-MS grade water to a final methanol concentration of 53%, transferred to fresh tubes, and centrifuged again under the same conditions [38]. The resulting supernatants were subjected to metabolite analysis.

UHPLC-MS/MS analysis was conducted by Novogene Co., Ltd. (Beijing, China) using a Vanquish UHPLC system (Thermo Fisher Scientific, Germering, Germany) coupled with an Orbitrap high-resolution mass spectrometer (Thermo Fisher Scientific, Germering, Germany). Metabolite separation was performed on a Hypersil Gold column (100 × 2.1 mm, 1.9 μm) with a mobile phase consisting of water containing 0.1% formic acid (A) and methanol (B). Elution was carried out at a flow rate of 0.2 mL min^−1^ using a linear gradient program as follows: 2% B for 1.5 min, increased to 85% B at 3 min, raised to 100% B at 10 min, returned to 2% B at 10.1 min, and maintained until 12 min. Mass spectrometry data were acquired under both positive and negative ionization modes. Instrument settings included a spray voltage of 3.5 kV, capillary temperature of 320 °C, sheath gas flow rate of 35 arb, auxiliary gas flow rate of 10 arb, S-lens RF level of 60, and auxiliary gas heater temperature of 350 °C.

### 2.7. Metabolomic Data Processing and Analysis

Metabolomic data acquisition was performed by Novogene Co., Ltd. (Beijing, China). Raw data generated from UHPLC–MS/MS were processed using XCMS for peak alignment, peak detection, and metabolite quantification [39,40]. Metabolite identification was performed by matching acquired spectra against the high-quality in-house secondary spectral database (NovoMetDB) developed by Novogene. This database integrates spectra acquired from authentic reference standards together with curated high-quality MS/MS spectra collected from public databases, including HMDB, RefMetaDB, and ReSpect for Phytochemicals.

All 42 metabolomic samples were analyzed within a single analytical batch, thereby minimizing potential inter-batch variation. Pooled quality control (QC) samples (prepared by mixing equal volumes of all samples) were injected periodically throughout the run to monitor instrument stability. Data preprocessing was subsequently performed by removing metabolites with more than 50% missing values across biological samples, followed by imputation of the remaining missing values using the K-nearest neighbor (KNN) algorithm. Background ions were removed according to blank samples, and QC-based normalization was applied to reduce analytical variation among samples. Metabolites with coefficients of variation (CV) greater than 30% in QC samples were excluded from subsequent analyses. Duplicate metabolite features were then removed based on InChIKey or compound name, retaining the annotation with the highest confidence level and matching score.

Metabolite annotation was performed according to the Metabolomics Standards Initiative (MSI) framework using the Novogene in-house metabolite database. For MSI Level 1 annotation, metabolite features were assigned by concordant matching of MS1 information, MS/MS spectra, and retention time (RT) with reference-standard-derived entries in the database. The reference standards used for database construction were analyzed under conditions consistent with those used for the present study. MSI Level 2 annotation was assigned when both MS1 information and MS/MS spectra matched entries in the database, whereas MSI Level 3 annotation was assigned based on matching of MS1 information and was considered a putative annotation at the compound-class level. The mass tolerance was set to 10 ppm for MS1 and 0.1 Da for MS/MS. Metabolite features detected in positive and negative ionization modes were subsequently integrated to generate the final metabolite dataset for downstream analyses.

Following metabolite identification, metabolites were further annotated using the KEGG (https://www.genome.jp/kegg/pathway.html, accessed on 10 February 2026), HMDB (https://hmdb.ca/metabolites, accessed on 10 February 2026), and LIPIDMaps (http://www.lipidmaps.org/ , accessed on 10 February 2026) databases. PCA and differential metabolite analyses were performed using R software and NovoMagic, an online analytical platform developed by Novogene. To characterize metabolic dynamics during development, stage-dependent analyses were performed using sequential developmental samples. KEGG pathway enrichment analysis was conducted based on differential metabolites. All metabolites annotated to KEGG pathways were used as the background population. The top 20 enriched pathways ranked by enrichment significance were selected for visualization and interpretation.

### 2.8. Transcriptome-Metabolome Cluster Correlation Analysis

To explore coordinated developmental patterns between the transcriptome and metabolome, cluster-level correlation analysis was performed separately for pileus and stipe tissues. Transcriptomic features were first assigned to six expression clusters and metabolomic features to three abundance clusters based on their temporal profiles across the four developmental stages (F1–F4). For each cluster, the mean expression (transcriptome) or mean abundance (metabolome) across biological replicates was calculated at each stage, generating a four-point developmental profile per cluster. These cluster profiles were then row-wise Z-score normalized to emphasize similarities in temporal pattern rather than differences in absolute magnitude. Pearson correlation coefficients were calculated between each transcriptomic cluster profile and each metabolomic cluster profile. Because the analysis was based on only four ordered developmental stages, the resulting correlation coefficients were interpreted primarily as measures of similarity in stage-dependent patterns, rather than as formal evidence of direct regulatory relationships.

## 3. Results

### 3.1. Environmental Temperature and Humidity Conditions During Off-Season Industrial Cultivation

Stable fruiting of *Morchella eximia* (strain G10) was successfully achieved under off-season industrial cultivation conditions in Hangzhou, China, during the summer–autumn period (September to November 2025). For the batch used in this study, cultivation was performed on a 70 m^2^ scale, yielding an average of 1.79 kg/m^2^. Through comparative analysis of environmental parameters from conventional in-season cultivation, key conditions required for off-season industrial cultivation were identified and precisely regulated, enabling stable fruiting body development.

**Figure 1:**
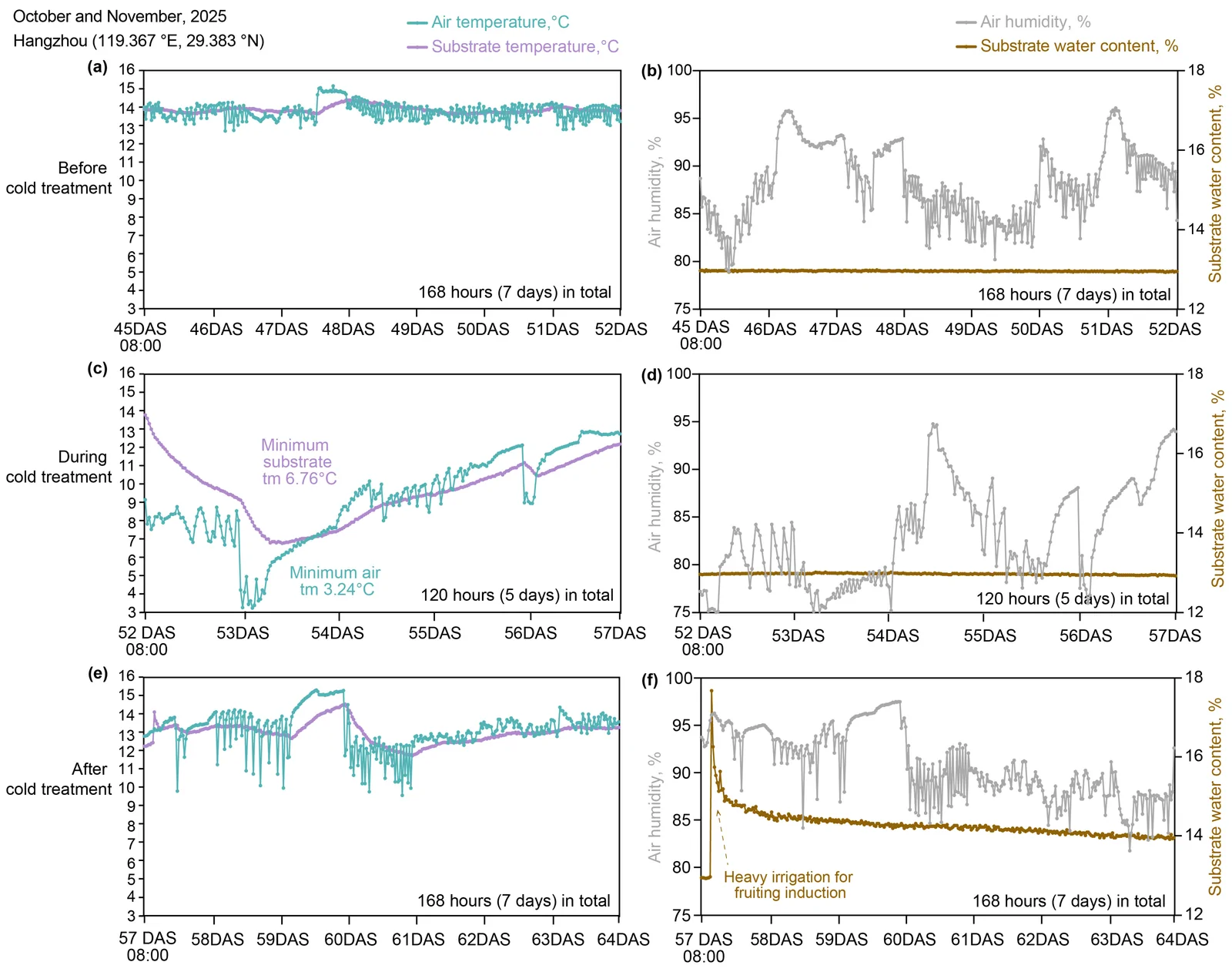
Environmental temperature and humidity dynamics during industrial cultivation of *M. eximia* G10. (**a**,**c**,**e**) Air temperature and substrate temperature recorded during different cultivation stages. (**b**,**d**,**f**) Air relative humidity and substrate moisture content recorded during the corresponding stages. (**a**,**b**) Mycelial cultivation stage; (**c**,**d**) low-temperature stimulation stage; (**e**,**f**) fruiting stage.

The industrial cultivation process of G10 was divided into three key stages: mycelial cultivation, low-temperature stimulation, and fruiting management. During the mycelial cultivation and fruiting management stages, both substrate and ambient temperatures were maintained at approximately 12–15 °C to support mycelial growth and fruiting body development (Figure 1a,e). After approximately 50 days of mycelial cultivation, the cultures were subjected to a low-temperature stimulation treatment at around 5 °C for 5 days to induce the transition from vegetative growth to reproductive development (Figure 1c). Following low-temperature stimulation, the temperature was restored to 12–15 °C, and the substrate was thoroughly irrigated for fruiting induction (Figure 1e).

Due to the high ambient temperatures during the cultivation period, fluctuations in environmental conditions within the cultivation rooms were unavoidable. The dynamic changes in substrate and air temperature throughout the cultivation process were continuously monitored and are presented in Figure 1. Although air temperature exhibited substantial fluctuations during cultivation, substrate temperature remained comparatively stable throughout the developmental process.

Relative air humidity was maintained within a target range of 75–95% during most stages of cultivation (Figure 1b,d,f). Following heavy irrigation, humidity levels rapidly increased to 97–98% for a short period before gradually stabilizing within the normal management range (Figure 1f). Under routine cultivation conditions, substrate moisture content remained relatively stable at 12.9–13.0% (Figure 1b,d,f). To support primordium differentiation and maintain proper fruiting body morphology, heavy irrigation was applied during the fruiting stage, resulting in a rapid increase in substrate moisture to 17.7%, followed by a gradual decline to approximately 14% during subsequent development (Figure 1f).

Overall, the cultivation system maintained a relatively stable and controllable microenvironment, under which successful off-season fruiting of *M. eximia* was achieved despite the high-temperature seasonal conditions. These observations indicate that controlled regulation of environmental temperature and humidity was associated with successful fruiting under the off-season cultivation conditions used in this study. The stable industrial cultivation system provided a valuable opportunity to investigate the growth and developmental processes of *M. eximia*.

### 3.2. Dynamic Transcriptomic Profiling Across Developmental Stages of G10

#### 3.2.1. Stage-Specific Sampling Reveals Developmental Transcriptomic Dynamics

**Figure 2:**
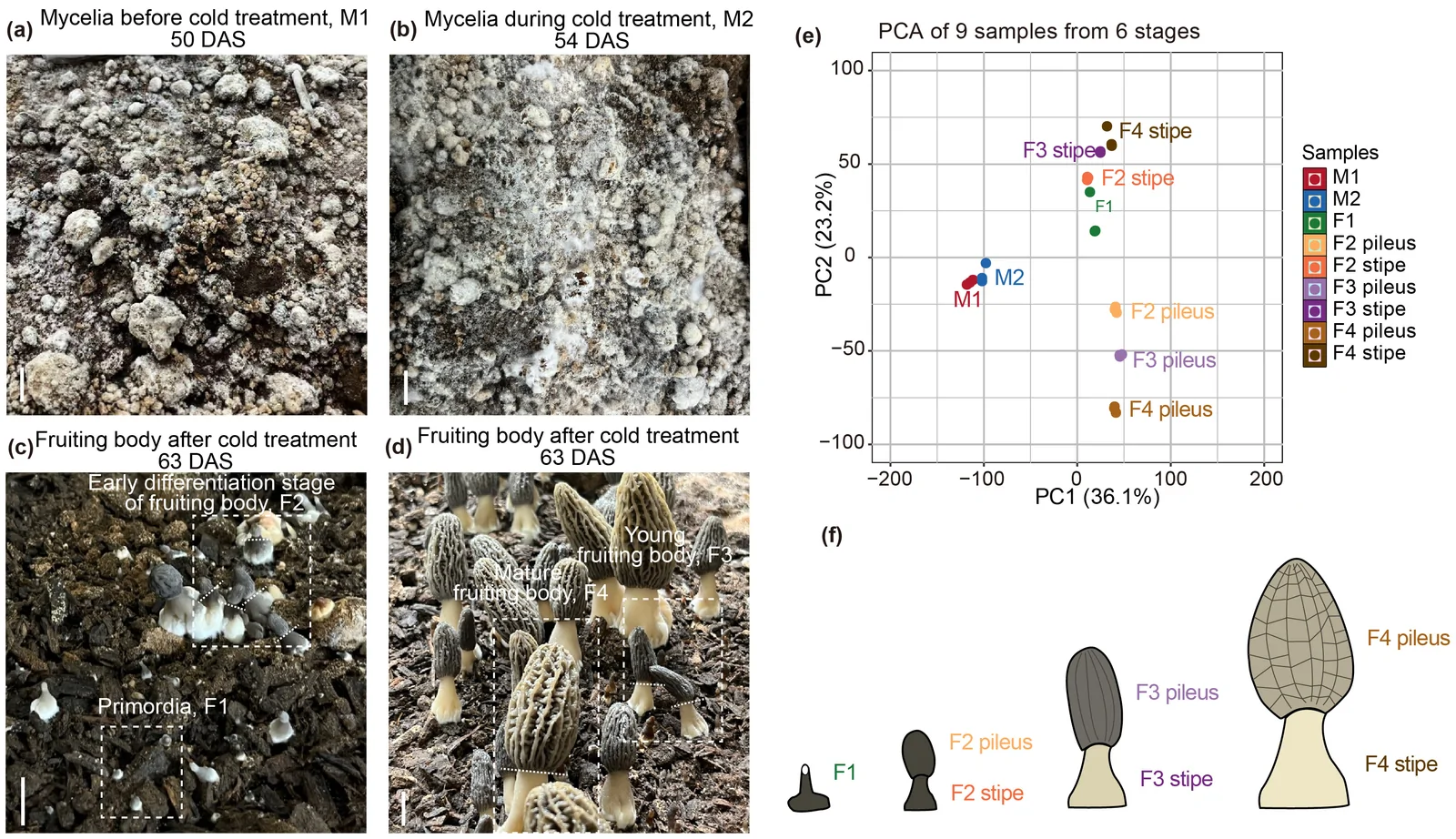
Sample collection and transcriptomic profiling across six developmental stages of G10. (**a**,**b**) Sampling of mycelial developmental stages, including pre-cold-treated (sampled at 50 DAS) and low-temperature-treated mycelia (sampled at 54 DAS). (**c**,**d**) Sampling of fruiting body developmental stages (sampled at 63 DAS). (**e**) PCA of transcriptomic data across different developmental stages and tissues. Each sample had three biological replicates. (**f**) Morphological developmental model of fruiting body formation in *M. eximia* G10.

Samples were collected at key developmental stages of G10, including pre-cold-treated mycelia (M1), low-temperature-treated mycelia (M2), primordia formed after low-temperature stimulation (F1), fruiting bodies at the initial pileus-stipe differentiation stage (F2), young fruiting bodies (F3), and mature fruiting bodies (F4) (Supplementary Table S1). Mycelial samples were carefully scraped from the substrate surface using a sterile spatula. Unlike previous studies that typically used mycelia cultured on Petri dishes, this study collected mycelia directly from the substrate surface to obtain more biologically relevant and realistic transcriptional profiles under industrial cultivation conditions. At the fruiting body developmental stages in which pileus and stipe tissues could be clearly distinguished (F2, F3, and F4), pileus and stipe tissues were collected separately for transcriptomic analysis (Figure 2a–d).

**Figure 3:**
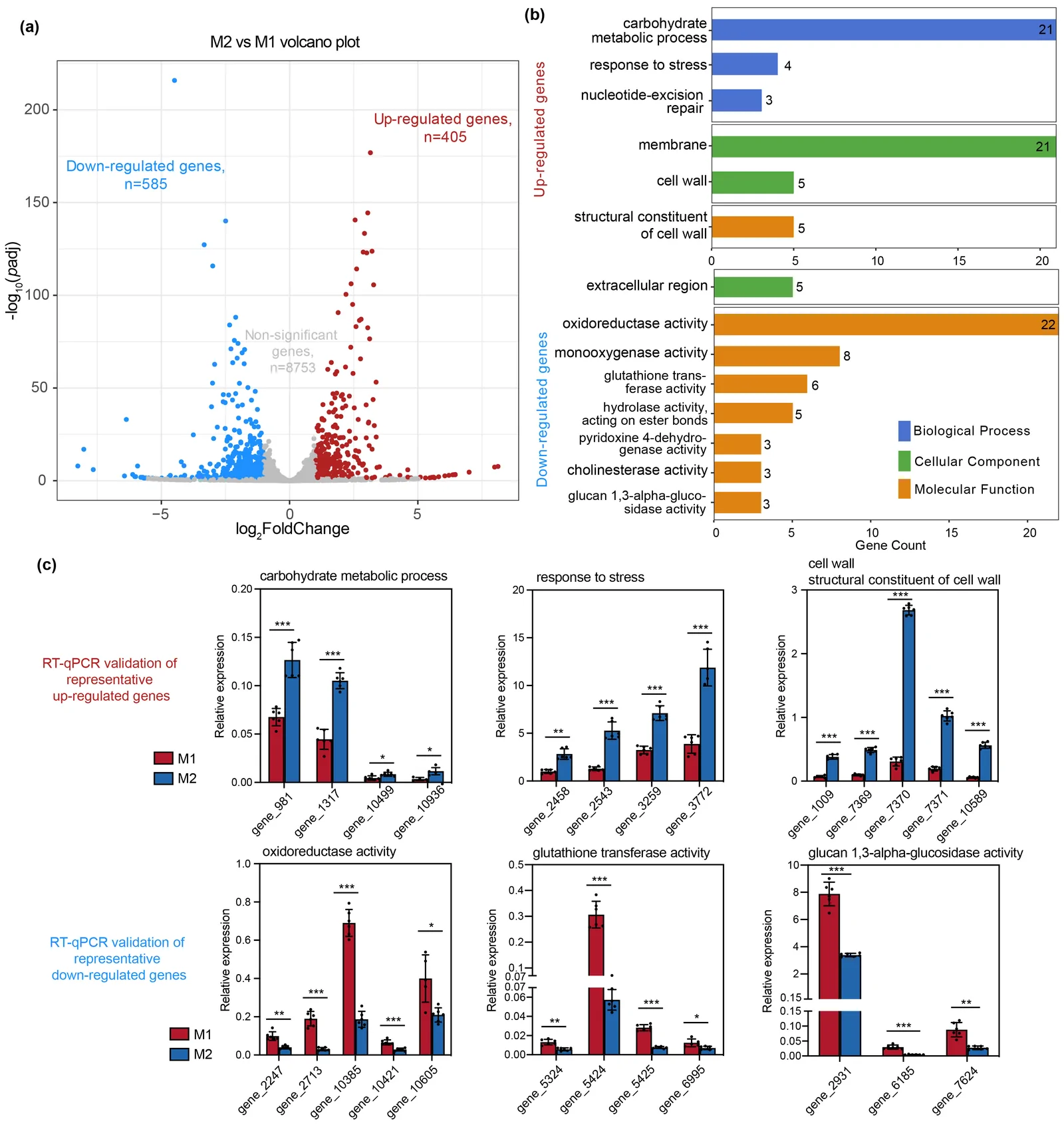
Transcriptomic comparison of mycelia before and after low-temperature stimulation in G10. (**a**) Volcano plot showing DEGs between pre- and post-low-temperature-treated mycelia. (**b**) GO enrichment analysis of DEGs, highlighting the biological processes, cellular components, and molecular functions significantly affected by low-temperature stimulation. (**c**) RT-qPCR validation of representative DEGs selected from enriched GO terms identified in M2 vs. M1. Statistical significance was determined using two-tailed Student’s *t*-test. * indicates *p* < 0.05, ** indicates *p* < 0.01, and *** indicates *p* < 0.001.

Principal component analysis (PCA) and sample correlation analysis both revealed clear transcriptional separation between mycelial and fruiting body stages (Figure 2e and Figure 3a). In addition, pileus and stipe tissues formed distinct clusters, indicating strong tissue-specific transcriptional regulation during fruiting body development. Interestingly, primordia clustered more closely with stipe tissues than with pileus tissues (Figure 2e, Supplementary Figure S1a), which may indicate that stipe tissues retain transcriptional features more similar to those of primordia. This observation raises the possibility that stipe differentiation may occur earlier than pileus during fruiting body development in *M. eximia*, although additional developmental and anatomical evidence will be required to test this hypothesis.

A developmental model illustration of fruiting body formation was also established (Figure 2f). During development, both pileus and stipe tissues gradually changed in color from dark black to yellow-brown. From the F3 to F4 stages, characteristic pits and ridges formed on the pileus surface, while the pileus morphology transitioned from a slender shape to a distinctly expanded basal structure.

We performed pairwise transcriptomic comparisons following the developmental trajectory from mycelial growth to pileus and stipe formation, resulting in a total of eight comparison groups (Supplementary Figure S1b, Supplementary Tables S3–S10).

The transition from low-temperature-treated mycelia to primordium formation exhibited the largest number of differentially expressed genes (DEGs), indicating a pronounced transcriptional reprogramming during this key developmental switch, which is consistent with the findings reported by *M. sextelata* [26]. As development progressed, both pileus and stipe tissues showed a gradual decrease in the number of differentially expressed genes in later stages, suggesting that transcriptional dynamics become progressively stabilized during fruiting body maturation. During the developmental trajectory from initial pileus-stipe differentiation to maturity, the pileus exhibited a more pronounced trend of gene upregulation, indicating active transcriptional activation associated with its continued morphogenesis. In contrast, the stipe developmental trajectory was characterized by a markedly stronger trend of gene downregulation, suggesting progressive transcriptional attenuation as stipe development proceeded toward maturation (Supplementary Figure S1c).

#### 3.2.2. Transcriptional Changes Associated with Cold Treatment During Primordium Initiation

To investigate how low-temperature stimulation triggers the transition from vegetative growth to reproductive development, we compared transcriptomes of mycelia before (M1) and after (M2) cold treatment.

Cold treatment was associated with substantial transcriptional reprogramming, resulting in 990 differentially expressed genes (DEGs), including 405 upregulated and 585 downregulated genes (Figure 3a, Supplementary Table S3). In addition, many DEGs exhibited large expression changes (|log_2_FC| > 5), indicating strong and specific transcriptional responses to low-temperature treatment.

Up-regulated genes were mainly associated with cell wall-related functions, carbohydrate metabolism, and stress responses, suggesting enhanced cellular remodeling and metabolic adjustment following cold exposure. In contrast, down-regulated genes were enriched in oxidoreductase and hydrolase-related functions, including processes involved in glucan degradation and other hydrolytic activities (Figure 3b,c).

Collectively, these transcriptional changes were associated with cellular remodeling and metabolic readiness during the transition from vegetative growth toward reproductive development, highlighting molecular changes accompanying the cold-treatment stage in mycelia. These transcriptional adjustments show partial convergence with cold-induced responses previously reported in *Pleurotus tuoliensis* and *Flammulina velutipes* [29,30,31]. Notably, regulation of cell wall-related processes and carbohydrate metabolism appears to be a shared and core feature accompanying the transition from vegetative growth to reproductive development across several edible fungi.

**Figure 4:**
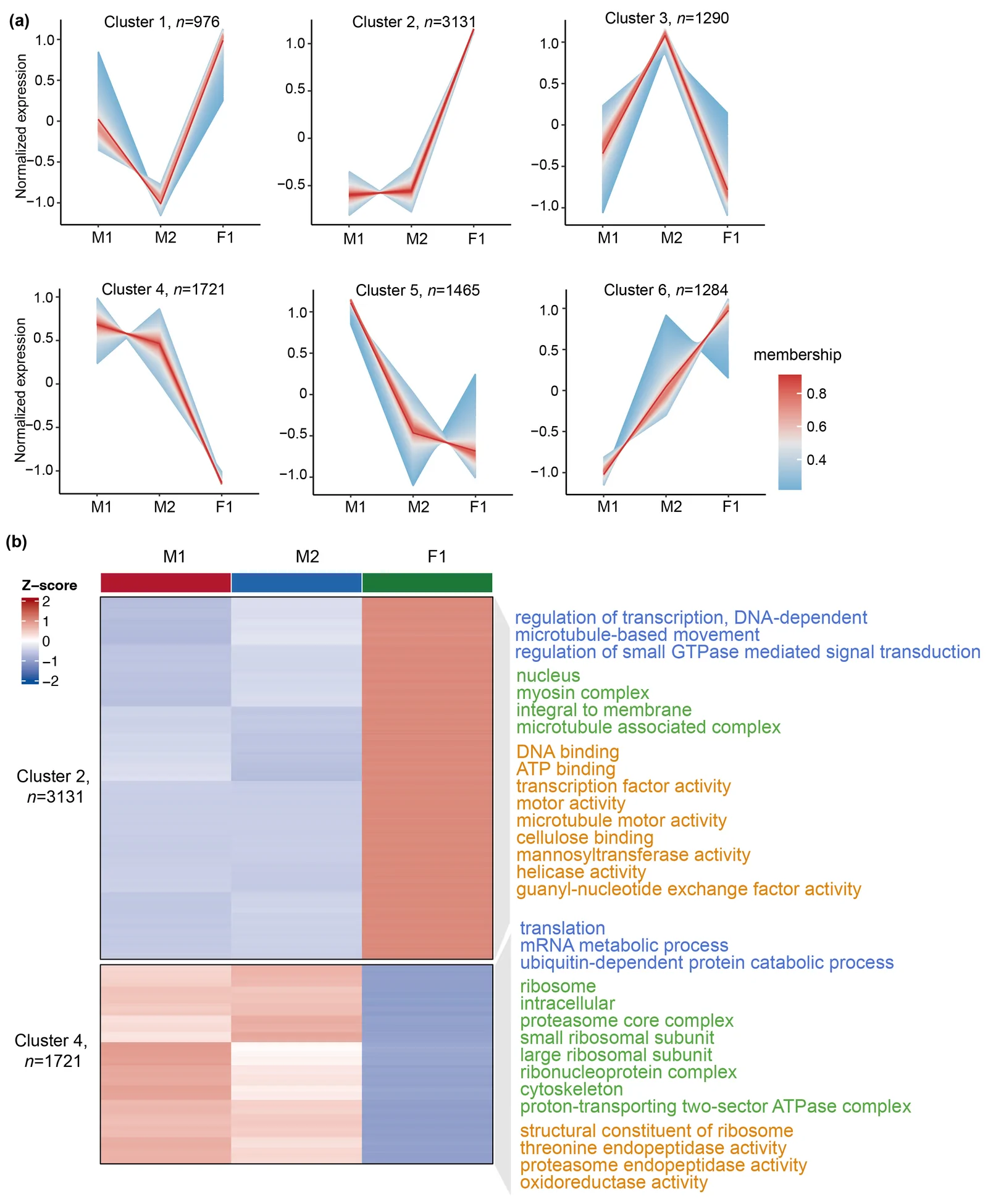
Stage-dependent transcriptomic analysis of mycelia before and after low-temperature stimulation and primordia formation in G10. (**a**) K-means clustering analysis of DEGs, which were classified into six distinct expression clusters. (**b**) Heatmaps showing row-wise Z-score normalized TPM values of representative clusters 2 and 4, together with GO enrichment analyses of the corresponding gene sets. Blue indicates BP, green indicates CC, and orange indicates MF.

To characterize the transcriptional dynamics associated with the transition from vegetative growth to primordium formation in *M. eximia*, stage-dependent expression profiling was performed across pre-cold mycelia, post-cold mycelia and primordia. DEGs were grouped into six distinct expression clusters via K-means clustering (Figure 4a, Supplementary Figure S2, Supplementary Table S11). Among these, cluster 2 (*n* = 3131) and cluster 4 (*n* = 1721) displayed the most pronounced stage-specific patterns.

Genes in cluster 2 remained at relatively low levels during the mycelial stages but were strongly induced upon primordium formation (Figure 4b). This induction coincided with the activation of transcriptional regulation, intracellular signaling, and cytoskeletal and membrane-related processes, suggesting that primordium initiation is accompanied by enhanced regulatory activity and extensive cellular reorganization.

In contrast, genes in cluster 4 showed high expression during the mycelial stages but were downregulated in primordia, suggesting reduced activity of pathways associated with mycelial maintenance (Figure 4b). These genes were mainly linked to protein synthesis, RNA metabolism, protein turnover, and energy metabolism. Their coordinated downregulation suggests a reduction in basic metabolic and biosynthetic activities, consistent with a shift from mycelial maintenance toward a developmental state supporting reproductive transition.

#### 3.2.3. Distinct Transcriptional Programs During Pileus and Stipe Development

**Figure 5:**
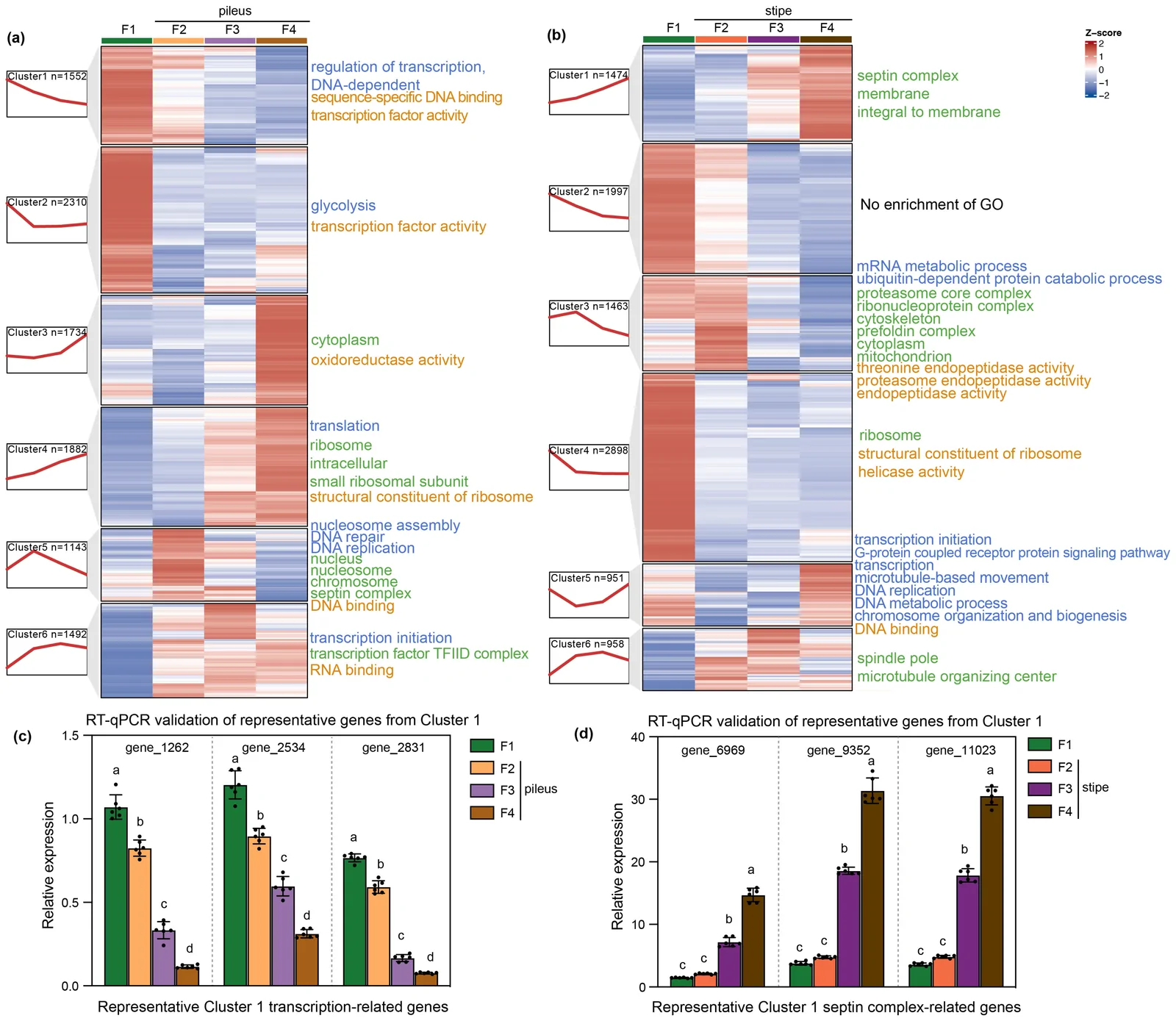
Stage-dependent transcriptomic analysis of pileus and stipe development in G10. Developmental transcriptomic profiling of the pileus (**a**) and stipe (**b**). For each panel, the left section shows the average expression trajectories of genes within each cluster, the middle section presents heatmaps of row-wise Z-score normalized TPM values, and the right section shows GO enrichment analysis of the corresponding gene clusters. Blue indicates BP, green indicates CC, and orange indicates MF. (**c**,**d**) RT-qPCR validation of representative genes from cluster 1 in the pileus (**c**) and stipe (**d**). Three representative transcription-related genes were selected for validation in the pileus, and all three were commonly annotated with the GO terms regulation of transcription, DNA-dependent, sequence-specific DNA binding, and transcription factor activity. In the stipe, three representative septin complex-related genes were selected for validation. Statistical significance among developmental stages was determined using one-way ANOVA followed by Tukey’s multiple comparison test. Different letters indicate significant differences (*p* < 0.05).

To investigate tissue-specific developmental regulation during fruiting body formation, stage-resolved clustering analysis was performed separately for pileus and stipe tissues across four developmental stages (F1-F4). DEGs were classified into six expression clusters, highlighting distinct transcriptional patterns associated with tissue development and maturation (Figure 5, Supplementary Tables S12 and S13).

In the pileus, gene expression patterns indicated a stage-dependent shift from early developmental regulation to later metabolic specialization (Figure 5a). Genes in clusters 1 and 2 showed relatively high expression during the early stages of primordium-to-pileus development and declined during maturation, whereas genes in clusters 3 and 4 displayed the opposite trend and showed progressively higher expression in mature tissues.

Early-expressed genes were predominantly linked to regulatory functions, particularly transcription-associated processes. RT-qPCR validation of three representative transcription-related genes from cluster 1 confirmed expression patterns consistent with the transcriptomic data (Figure 5c). In contrast, genes showed higher expression at later stages were more closely associated with protein biosynthesis and oxidative metabolism, consistent with a transition from tissue establishment and differentiation toward physiological maturation. Cluster 5 exhibited transient induction during the intermediate developmental stage (F2), suggesting a transient increase in expression of cell-cycle- and cytoskeleton-related functions during early pileus differentiation. Genes in cluster 6 remained weakly expressed in primordia but showed progressively higher expression after tissue differentiation, suggesting sustained activation of transcription- and RNA-related functions throughout pileus maturation.

Compared with the pileus, the stipe displayed a markedly different stage-dependent expression pattern (Figure 5b). Cluster 1 genes showed progressive activation throughout development, and the expression patterns of three representative septin complex-associated genes further validated by RT-qPCR (Figure 5d), supporting the robustness of this transcriptional pattern. In contrast, genes in Clusters 2, 3, and 4 exhibited lower expression across the developmental stages following primordium formation. Rather than reflecting isolated pathway changes, these profiles indicate a developmental shift in which the early stages of the primordium-to-stipe series were associated with cytoskeletal organization, energy metabolism, and protein turnover, whereas several programs related to protein synthesis and basic cellular metabolism became less prominent during later development. In addition, cluster 6 displayed elevated expression during the F2-F3 stages and was linked to microtubule-organizing center- and cytoskeleton-related functions, consistent with ongoing cytoskeletal organization during stipe growth and elongation. Unlike the predominantly declining expression patterns observed in several stipe clusters, cluster 5 exhibited a biphasic profile, with elevated expression during both the earliest and latest developmental stages. Its enrichment in transcriptional regulation, DNA-related processes, and signaling functions suggests that regulatory activities may be involved at both early differentiation and late maturation, although their specific roles remain to be determined.

Overall, the stipe exhibited a developmental program characterized by early activation of cytoskeletal and energy-related processes followed by a relative reduction in protein synthesis and basic cellular metabolic programs, together with stage-specific regulatory activity. In contrast, the pileus showed progressive activation of metabolic and translational pathways during maturation. These contrasting expression patterns support the distinct morphological and functional roles of the pileus and stipe in the fruiting bodies of *M. eximia*.

### 3.3. Tissue-Specific Metabolic Programs During Pileus and Stipe Development

**Figure 6:**
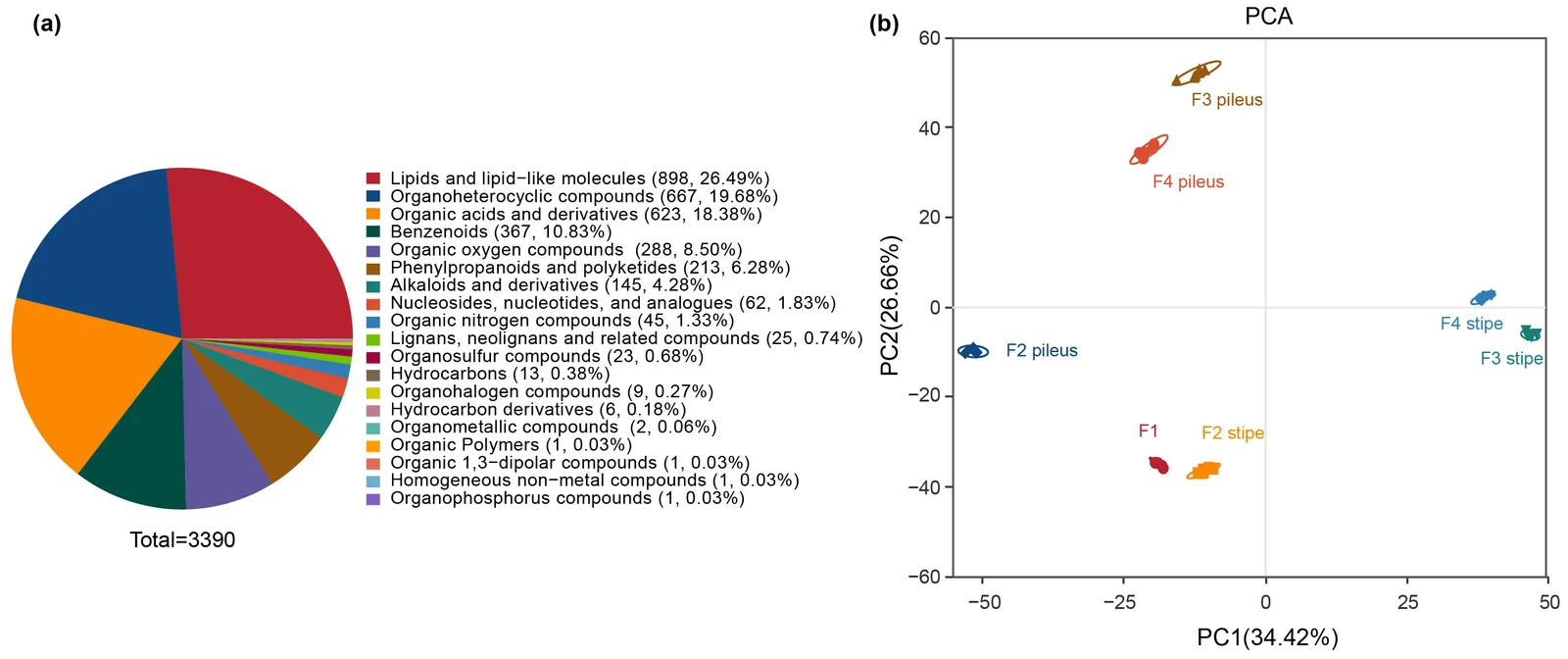
Metabolomic profiling of G10 pileus and stipe across developmental stages. (**a**) Classification of all annotated metabolites into chemical classes, with the number and percentage of metabolites in each category indicated. (**b**) PCA score plot showing the separation of metabolomic profiles across different developmental stages and tissues. The first two principal components (PC1 and PC2) explained 34.42% and 26.66% of the total variance, respectively. Each sample had six biological replicates for untargeted metabolomic analysis.

Because substrate particles and soil residues in the mycelial samples could interfere with metabolite extraction and detection, metabolomic profiling was not performed for mycelial stages. Therefore, metabolomic analyses were focused on fruiting body developmental stages. Untargeted metabolomic profiling detected a total of 3456 metabolite features, which were annotated at different confidence levels according to the MSI framework, with 3390 features successfully assigned to major chemical classes (Figure 6a, Supplementary Table S15), representing substantially higher metabolite coverage than previously reported studies in *Morchella* [26,27]. According to the Metabolomics Standards Initiative (MSI) framework, 401 metabolites were classified as Level 1, 1376 as Level 2, and 1779 as Level 3. PCA revealed clear separation among samples according to both developmental stage and tissue type, suggesting strong stage- and tissue-dependent metabolic variation (Figure 6b). Lipids and lipid-like molecules, organic acids and derivatives, and organoheterocyclic compounds were the three major metabolite classes, accounting for a large proportion of the detected metabolites.

**Figure 7:**
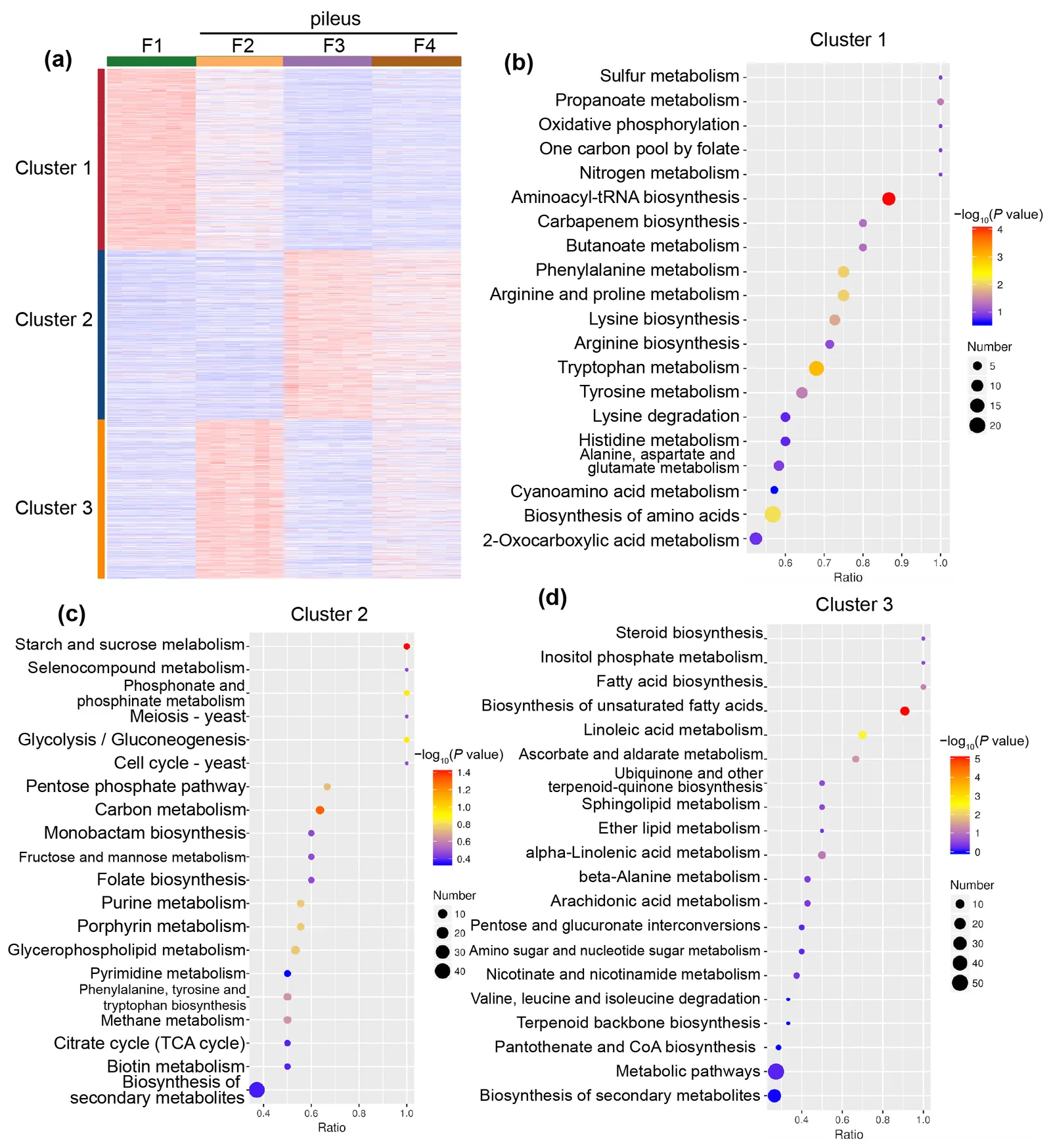
Metabolite clustering and KEGG pathway enrichment during pileus development of G10. (**a**) Heatmap showing three distinct stage-dependent metabolite clusters identified by K-means clustering based on row-wise Z-score normalized metabolite abundance across developmental stages. (**b**–**d**) KEGG enrichment of cluster 1 to 3.

In the pileus, three major metabolic clusters displaying distinct stage-dependent patterns were identified (Figure 7a). Metabolome cluster 1 showed positive associations with transcriptome clusters 1 and 2 (Pearson’s r = 0.94 and 0.92, respectively), suggesting a close correspondence between transcriptional and metabolic changes during the early stages of the primordium-to-pileus developmental series (Supplementary Figures S3a and S4a, Supplementary Table S16). Metabolites in this cluster showed their highest abundance predominantly at the primordium stage and were mainly associated with amino acid- and nitrogen-related metabolic processes (Figure 7a,b). To further examine this association, genes from transcriptome clusters 1 and 2 and metabolites from metabolome cluster 1 that shared KEGG pathway annotations were compared. The resulting profiles revealed coordinated stage-dependent patterns in pathways related to amino acid metabolism, energy metabolism, and sulfur metabolism (Supplementary Figure S5). These coordinated changes suggest that early pileus differentiation is associated with coordinated shifts in nitrogen metabolism, carbon skeleton supply, energy metabolism, and sulfur metabolism, potentially supporting the metabolic requirements associated with primordium establishment. Metabolites in cluster 2 showed higher abundance predominantly during the young and mature fruiting body stages, whereas cluster 3 peaked at the early differentiated stage and remained relatively elevated in mature tissues (Figure 7a). Functional enrichment suggested associations with central carbon metabolism in cluster 2 and lipid- and sterol-associated metabolic processes, in cluster 3 (Figure 7c,d). These patterns suggest a stage-dependent metabolic shift during pileus development, from early amino acid-focused biosynthesis toward enhanced carbon metabolism and later lipid-related activities.

**Figure 8:**
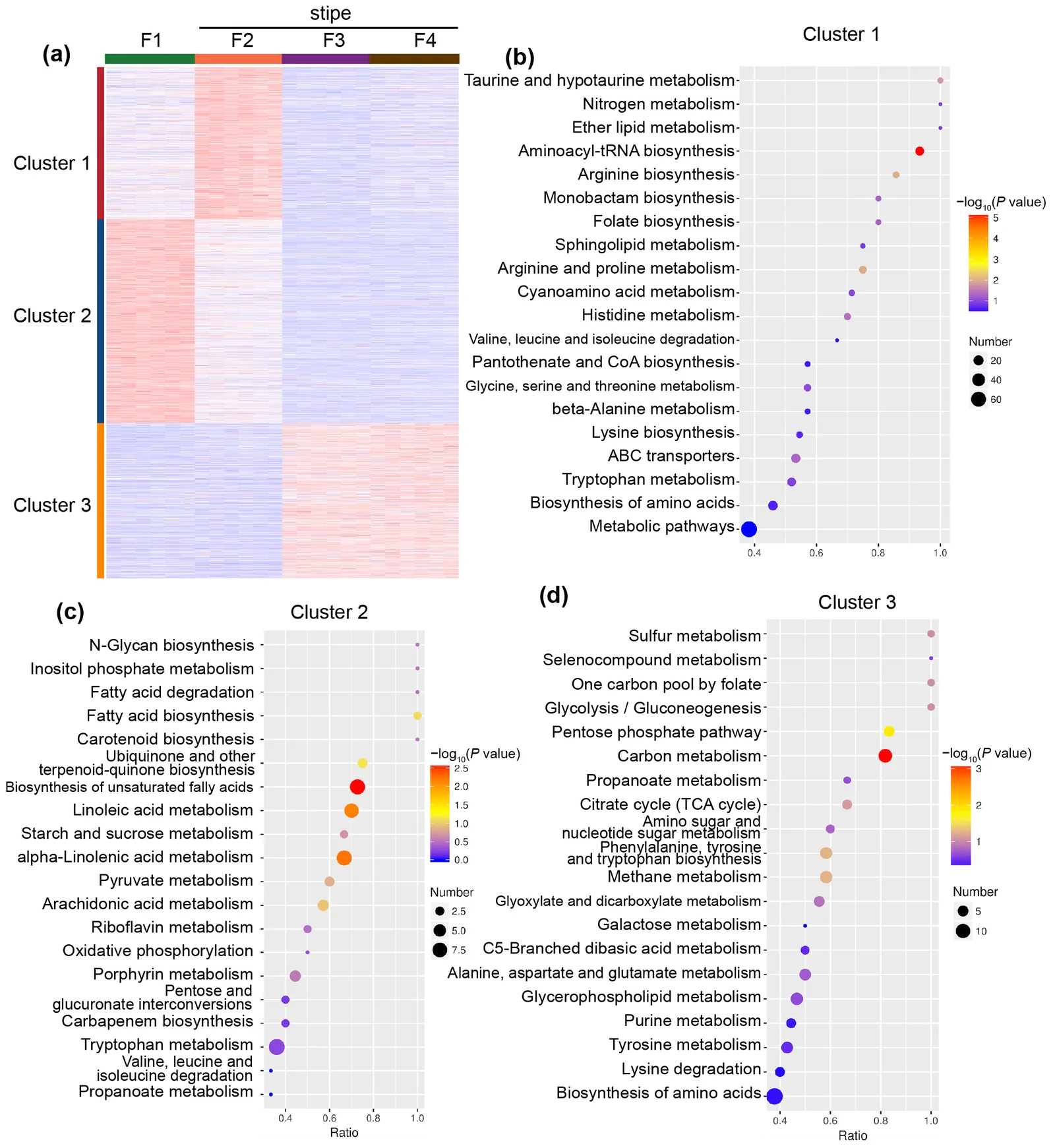
Metabolite clustering and KEGG pathway enrichment during stipe development of G10. (**a**) Heatmap showing three distinct stage-dependent metabolite clusters identified by K-means clustering based on row-wise Z-score normalized metabolite abundance across developmental stages. (**b**–**d**) KEGG enrichment of cluster 1 to 3.

Distinct metabolic trajectories were also observed in the stipe (Figure 8a). Metabolome cluster 1 reached its highest abundance at the F2 stage and declined thereafter, and showed a positive association with transcriptome cluster 3 (Pearson’s r = 0.86) (Supplementary Figures S3b and S4b, Supplementary Table S16). The shared KEGG pathway of aminoacyl-tRNA biosynthesis displayed highly coordinated stage-dependent patterns (Supplementary Figure S6). These genes encoded multiple aminoacyl-tRNA synthetases, while the corresponding amino acids accumulated in parallel. The enrichment of aminoacyl-tRNA biosynthesis during the early stages of the primordium-to-stipe developmental series, together with the accumulation of corresponding amino acids, may reflect increased requirements for protein production at this stage. Cluster 2 showed the highest abundance at the primordium stage and declined thereafter, which was mainly associated with fatty acid metabolism and related lipid processes (Figure 8c). In contrast, cluster 3 displayed relatively low abundance during early development but was markedly increased during the later stages (Figure 8a). This later-activated cluster was enriched in central carbon metabolism, sulfur metabolism and glycerophospholipid remodeling (Figure 8d). Taken together, the stipe showed an early metabolic emphasis on fatty acid metabolism and aminoacyl-tRNA biosynthesis, followed by a later shift toward central carbon metabolism, sulfur metabolism and lipid remodeling during maturation.

These contrasting trajectories indicate that amino acid-related metabolism represents a common metabolic feature of early development in both pileus and stipe. Nevertheless, the two tissues diverge in their subsequent metabolic priorities in accordance with tissue-specific developmental characteristics. The pileus shifted toward biosynthetic and energy-related processes associated with tissue differentiation and maturation, potentially reflecting increased metabolic demands during tissue specialization and reproductive development, whereas the stipe showed stronger signatures of aminoacyl-tRNA biosynthesis and translational demand during early development, followed by changes in carbon metabolism, sulfur metabolism, and lipid remodeling accompanying later maturation.

## 4. Discussion

### 4.1. Environmental Regulation and Developmental Dynamics of Morchella eximia Under Off-Season Industrial Cultivation

Temperature and humidity have consistently been regarded as major environmental factors affecting both vegetative mycelial growth and fruiting body formation in *Morchella* [41,42,43,44]. Under conventional cultivation, fruiting generally occurs after prolonged exposure to winter low temperatures followed by warming in spring. Our controlled cultivation system reproduced this environmental sequence by applying short-term low-temperature stimulation followed by restoration of favorable temperature and humidity conditions, supporting the view that low temperature is closely associated with the vegetative-to-reproductive transition, whereas subsequent environmental conditions support primordium differentiation and fruiting body development. This strategy enabled stable off-season fruiting and provided a controllable platform for dissecting the molecular basis of environmentally regulated development in *Morchella*.

Off-season industrial cultivation provides an opportunity to extend morel production beyond the conventional cultivation season and may contribute to a more consistent market supply during periods of high demand. Because fresh morels command premium market prices during the off-season, successful industrial cultivation has considerable commercial potential despite its higher production costs. The reproducibility of the cultivation strategy was further supported by three independent industrial cultivation trials of *M. eximia* G10, which showed consistent fruiting performance and comparable yields (Supplementary Table S17). However, practical application remains constrained by the energy and infrastructure requirements associated with precise environmental control. Future efforts should therefore focus on optimizing environmental regulation to reduce energy consumption and production costs while maintaining stable fruiting performance.

**Figure 9:**
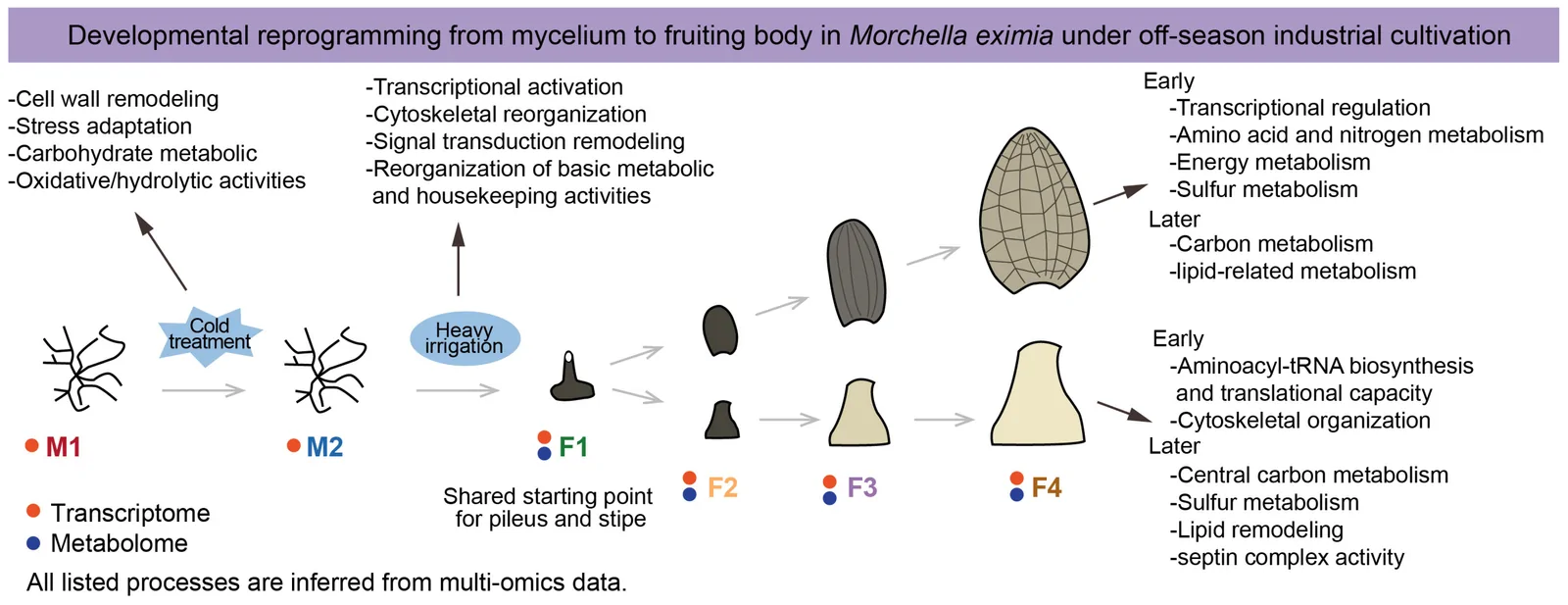
Proposed model illustrating off-season industrial cultivation and the coordinated transcriptomic and metabolic changes associated with *Morchella eximia* development. All processes shown are inferred from stage-dependent transcriptomic and metabolomic analyses. Potential regulatory relationships are hypothetical and require future functional validation.

Importantly, the controlled cultivation system allowed continuous sampling across developmental stages and separate analysis of pileus and stipe tissues, providing a basis for linking environmental transitions with stage- and tissue-specific transcriptional and metabolic reprogramming (Figure 9, Supplementary Table S18). Beyond demonstrating the feasibility and reproducibility of off-season fruiting, this study provides a valuable developmental molecular resource for *M. eximia* under industrial cultivation conditions. The cultivation framework and multi-omics dataset established here provide an important foundation for further functional studies and for optimizing environmental regulation toward more stable and potentially year-round industrial production of *Morchella*.

### 4.2. Cold-Associated Transcriptional Changes During Fruiting Body Formation in Morchella eximia

Because cold treatment was applied at a defined stage of vegetative development, these transcriptional changes may reflect the combined influence of low-temperature exposure and concurrent developmental progression. In the present study, pronounced transcriptional changes related to cell wall and membrane processes were observed following cold treatment. These responses are consistent with cold-induced adjustments previously reported in *Flammulina velutipes* and *Pleurotus eryngii* subsp. *Tuoliensis*, suggesting that cell wall and membrane remodeling may represent a partially conserved feature accompanying developmental progression under low-temperature conditions in mushroom-forming fungi [29,31]. Notably, a MAPK pathway-associated gene (gene_77) was significantly upregulated (approximately two-fold) after cold exposure (Supplementary Table S3), further supporting the possible involvement of MAPK-related signaling in this transition, in line with findings in other edible mushrooms [29,31]. The downregulation of genes associated with oxidoreductase- and hydrolase-related functions may represent additional features of the vegetative-to-reproductive transition in *Morchella*.

Stage-dependent transcriptomic analysis further showed that the transition from post-cold mycelia to primordia represented the most dramatic developmental shift during the entire life cycle. This pronounced reorganization of gene expression suggests that primordium initiation may correspond to a critical developmental checkpoint during which transcriptional profiles shift from those characteristics of vegetative mycelia toward those associated with reproductive development. Similar transitions have been reported in other mushroom-forming fungi, supporting the view that fruiting body initiation is accompanied by coordinated remodeling of cellular activities in response to developmental and environmental cues [30,45,46]. Although the present data do not establish direct regulatory relationships, they identify candidate biological processes that may contribute to cold-responsive developmental transitions in *Morchella*.

### 4.3. Transcriptional and Metabolic Reprogramming Underlying Fruiting Body Development in Morchella eximia

An important finding of this study is the tissue-specific nature of transcriptional and metabolic reprogramming during fruiting body development. PCA showed that tissue type had a greater impact on overall gene expression patterns than developmental stage, resulting in clear separation between pileus and stipe samples throughout fruiting body maturation. Tissue-specific transcriptional and metabolic specialization has also been reported in other edible fungi. In the present study, F1 was used as a common starting point for both pileus and stipe developmental trajectories because the two tissues were not yet distinguishable at this stage. Nevertheless, F1 represents the shared developmental starting point for subsequent pileus and stipe differentiation.

In *Stropharia rugosoannulata*, tissue-resolved transcriptomic analysis revealed preferential amino acid metabolism in the pileus and sugar metabolism in the stipe [47]. Meanwhile, early fruiting body development in *Agaricus bisporus* is characterized by cell wall remodeling and glycoside hydrolase activity [48]. In *M. eximia*, both amino acid and carbohydrate metabolism were prominent features of pileus and stipe development, although their relative contributions varied across developmental stages. In *Lentinula edodes*, Heat shock protein 70 (HSP70) has been implicated in pileus development. Similarly, five HSP70 homologs (gene_6343 in cluster 4, gene_1358 in cluster 6, gene_7588, gene_2804 and gene_11034 in cluster 3) showed predominantly later-stage expression in the *M. eximia* pileus [49], suggesting that protein homeostasis and stress adaptation may be recurrent features of pileus maturation across species. In addition, coordinated accumulation of amino acid-related metabolites and expression of genes involved in the corresponding metabolic pathways during early stages of the primordium-to-pileus developmental series suggests that amino acid and nitrogen metabolism may be particularly important during tissue differentiation, followed by a shift toward carbon and lipid metabolism during later development.

In the stipe, early developmental stages were characterized by coordinated changes linked to protein synthesis and cellular organization. Aminoacyl-tRNA biosynthesis and the accumulation of corresponding amino acids were prominent, accompanied by increased expression of genes associated with cytoskeletal organization. These patterns are consistent with observations in *Flammulina filiformis*, where cell wall synthesis, fatty acid metabolism and ROS-associated signaling have been associated with stipe elongation [50,51]. As development progressed in *M. eximia*, the metabolic emphasis shifted toward central carbon metabolism, sulfur metabolism and glycerophospholipid remodeling, consistent with a transition from active growth to structural and physiological maturation.

Environmental signals are known to influence tissue differentiation in other mushrooms, including light, temperature and oxygen cues [47]. The tissue-resolved multi-omics framework established here therefore provides a basis for identifying candidate regulators and metabolic processes associated with the morphological and functional divergence of pileus and stipe during fruiting body development under controlled industrial conditions.

### 4.4. Study Limitations and Future Perspectives

Several limitations of this study should be acknowledged. First, all analyses were conducted using a single *M. eximia* strain G10 under a specific industrial cultivation system, and therefore the observed molecular patterns may not be fully representative of other *Morchella* species, strains, or cultivation conditions. The fruiting body stages were sampled simultaneously at 63 days after sowing due to asynchronous development within the cultivation system, and thus represent morphologically defined developmental stages rather than a strictly chronological series.

Second, the coordinated transcriptomic and metabolomic analyses were primarily descriptive, and the observed associations between gene expression and metabolite accumulation should not be interpreted as direct regulatory relationships. In the metabolomic KEGG enrichment analysis, some pathway-level signals did not reach conventional statistical significance but were retained as exploratory observations to provide a broader view of potential metabolic changes during fruiting body development. In addition, metabolomic profiling was limited to reproductive stages, whereas transcriptomic analysis covered all developmental stages, resulting in incomplete overlap between the two datasets. Consequently, metabolic changes during the vegetative-to-reproductive transition could not be fully resolved from the metabolomic perspective.

Although representative genes from several enriched pathways were validated by RT-qPCR in this study, comprehensive functional characterization of candidate genes was not performed. Therefore, the biological roles of specific genes and regulatory pathways identified through transcriptomic and metabolomic analyses remain to be experimentally validated. Comprehensive functional genomic studies were not performed in the present study, partly due to the limited availability of genetic manipulation tools and functional annotation resources in *Morchella* compared with model fungal systems.

Future studies should therefore focus on functional validation of key candidate genes identified here. Approaches including gene editing, RNA interference, overexpression, heterologous expression, and reverse-genetics analyses will be important for determining whether candidate genes play causal roles in reproductive development and tissue differentiation. Integration of proteomic data with multi-layer omics approaches may further improve the understanding of regulatory mechanisms underlying fruiting body development. Moreover, systematic environmental manipulation experiments involving temperature, humidity, and nutrient availability will be important for dissecting how external cues influence transcriptional and metabolic reprogramming in *Morchella*. Improved mycelial isolation procedures and metabolomic profiling of pre-reproductive stages will also help resolve metabolic changes associated with reproductive induction. Together, these approaches will extend the present developmental atlas from descriptive molecular patterns toward experimentally validated regulatory mechanisms and ultimately improve its value for understanding and manipulating Morchella development under industrial cultivation conditions.

Despite these limitations, the present work provides a valuable transcriptomic and metabolomic dataset spanning multiple developmental stages and tissues, contributing to a better understanding of fruiting body development in *Morchella* and supporting future mechanistic investigations.

## 5. Conclusions

In this study, we established a controllable off-season industrial cultivation system for *Morchella eximia* and characterized developmental reprogramming from vegetative mycelia to mature fruiting bodies. Following the cold-treatment stage, extensive transcriptional changes were observed, particularly involving cell wall functions, carbohydrate metabolism, stress responses, and oxidoreductase- and hydrolase-related activities. During fruiting body development, the pileus and stipe displayed distinct yet coordinated transcriptional and metabolic programs. Both tissues showed early changes in amino acid-related metabolism. The pileus exhibited coordinated transcriptional and metabolic patterns related to amino acid- and nitrogen-related processes during the early stages of the primordium-to-pileus developmental trajectory, followed by increased carbon and lipid-associated metabolism, whereas the stipe showed prominent aminoacyl-tRNA biosynthesis and associated amino acid accumulation patterns during the early stages of the primordium-to-stipe developmental trajectory, followed by adjustments in carbon, sulfur and lipid metabolism during maturation. These findings provide a molecular framework for understanding environmentally regulated and tissue-specific developmental processes in M. eximia under controlled off-season cultivation, and provide a basis for future functional validation and cultivation optimization.

## Data Availability

The raw transcriptome sequencing data generated in this study have been deposited in the NCBI Gene Expression Omnibus (GEO) database with the accession number GSE333452. The metabolomics data generated in this study have been deposited in the China National Center for Bioinformation database with the accession number OMIX020107.

## Supplementary Materials

The following supporting information can be downloaded at: https://www.mdpi.com/article/10.3390/jof12090665/s1, Supplementary Figure S1. Sample correlation analysis and transcriptome comparison groups across developmental stages of G10. Supplementary Figure S2. GO enrichment analysis of Clusters 1-6 across the M1, M2, and F1 developmental stages. Supplementary Figure S3. Correlation analysis between transcriptome clusters and metabolome clusters in pileus and stipe tissues of *Morchella eximia*. Supplementary Figure S4. KEGG enrichment analysis of selected transcriptome clusters in pileus and stipe tissues. Supplementary Figure S5. Integrated transcriptomic and metabolomic analysis of shared KEGG pathways in pileus tissue. Supplementary Figure S6. Integrated transcriptomic and metabolomic analysis of shared KEGG pathways in stipe tissue.Supplementary Table S1. Sample information for transcriptomic and metabolomic analyses. Supplementary Table S2. Summary of RNA-seq quality metrics. Supplementary Table S3. DEGs between M2 vs M1. Supplementary Table S4. DEGs between F1 vs M2. Supplementary Table S5. DEGs between F2 pileus vs F1. Supplementary Table S6. DEGs between F3 pileus vs F2 pileus. Supplementary Table S7. DEGs between F4 pileus vs F3 pileus. Supplementary Table S8. DEGs between F2 stipe vs F1. Supplementary Table S9. DEGs between F3 stipe vs F2 stipe. Supplementary Table S10. DEGs between F4 stipe vs F3 stipe. Supplementary Table S11. Gene lists of expression clusters across M1, M2, and F1 stages. Supplementary Table S12. Gene lists of expression clusters across F1, F2, F3 and F4 pileus. Supplementary Table S13. Gene lists of expression clusters across F1, F2, F3 and F4 stipe. Supplementary Table S14. Gene-specific primer sequences used for RT-qPCR. Supplementary Table S15. Annotation information for all detected metabolites identified by UHPLC–MS/MS analysis. Supplementary Table S16. Pearson correlation statistics between transcriptomic and metabolomic cluster profiles across reproductive developmental stages in pileus and stipe tissues. Supplementary Table S17. Reproducibility of off-season industrial cultivation of *Morchella eximia* G10. Supplementary Table S18. Summary of major transcriptional and metabolic features during key developmental transitions in *Morchella eximia*.
