# Supplementary material for: Environmentally Associated Developmental Reprogramming from Mycelial Growth to Fruiting Body Formation in Morchella eximia Under Off-Season Industrial Cultivation
Source: J Fungi (Basel). 2026 Sep 3;12(9):665. doi: 10.3390/jof12090665 (PMC13608428; doi:10.3390/jof12090665)
Supplement: Supplementary file 1 [file jof-12-00665-s001.zip › Supplementary figure.pdf]

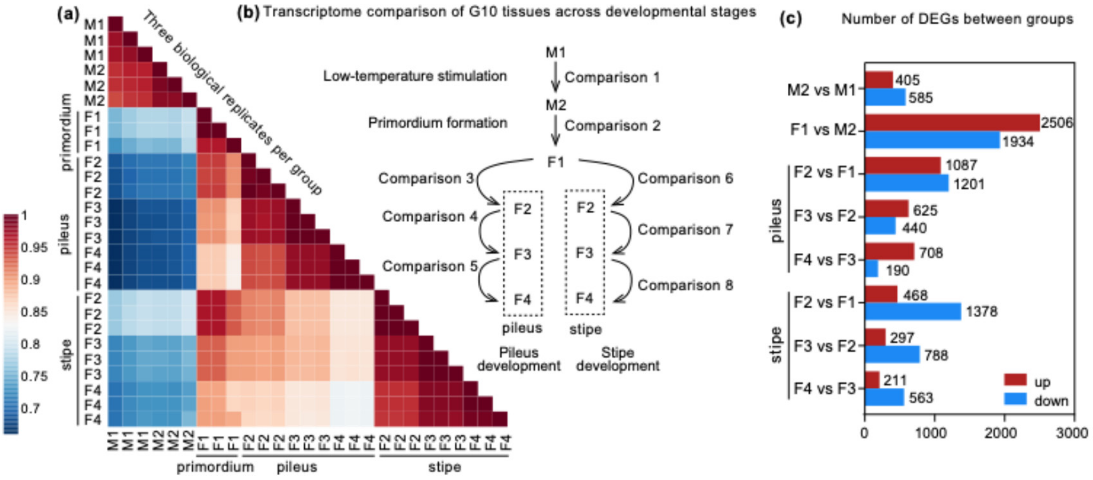

Supplementary Figure S1. Sample correlation analysis and transcriptome comparison groups across developmental stages of G10. (a) Correlation analysis among all samples based on transcriptomic profiles. (b) Schematic overview of eight pairwise comparison groups arranged according to developmental progression and tissues. (c) Number of DEGs corresponding to each comparison group shown in (b). The comparison between primordium formation and mycelial stage exhibited the highest number of DEGs, indicating a major transcriptional reprogramming during the transition from vegetative growth to reproductive development.

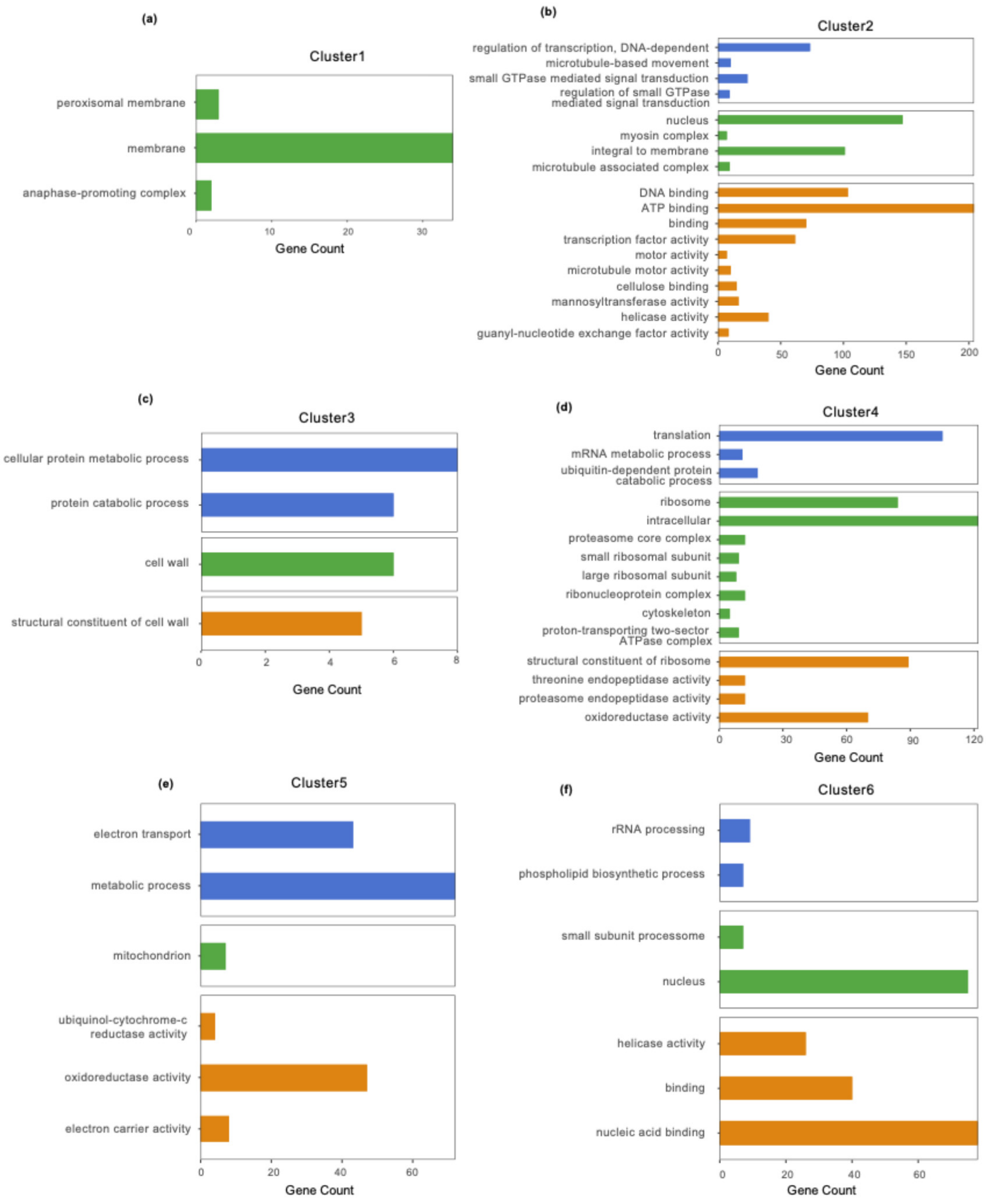

Supplementary Figure S2. GO enrichment analysis of Clusters 1-6 across the M1, M2, and F1 developmental stages.

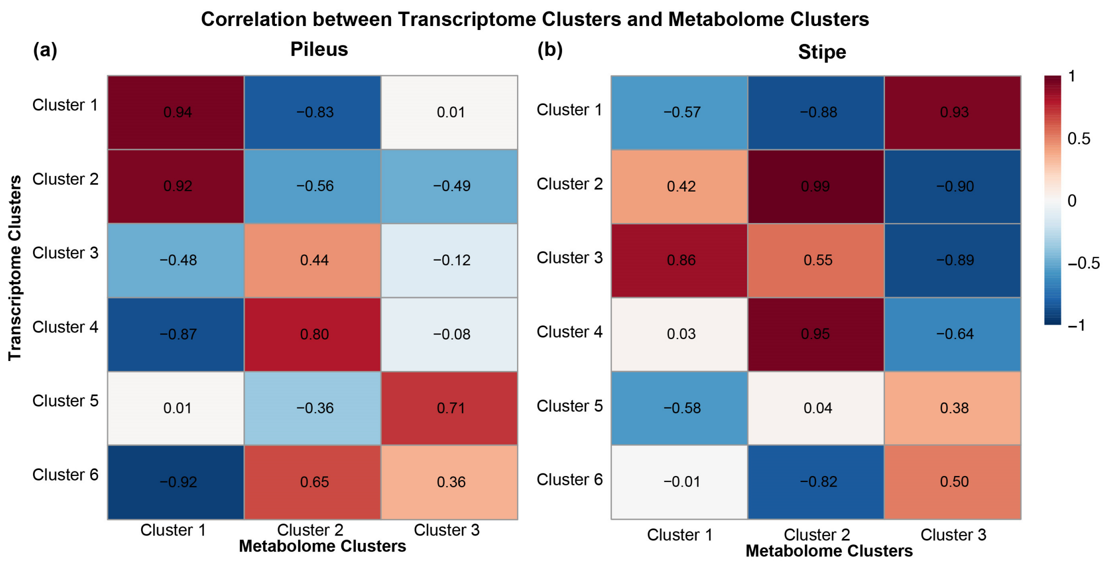

Supplementary Figure S3. Correlation analysis between transcriptome clusters and metabolome clusters in pileus and stipe tissues of *Morchella eximia*. Correlation heatmap for pileus tissues (a) and stipe tissues (b). Color scale indicates the Pearson correlation coefficient between the average expression profiles of transcriptome clusters (rows) and metabolome clusters (columns), ranging from –1 (strong negative correlation, blue) to +1 (strong positive correlation, red).

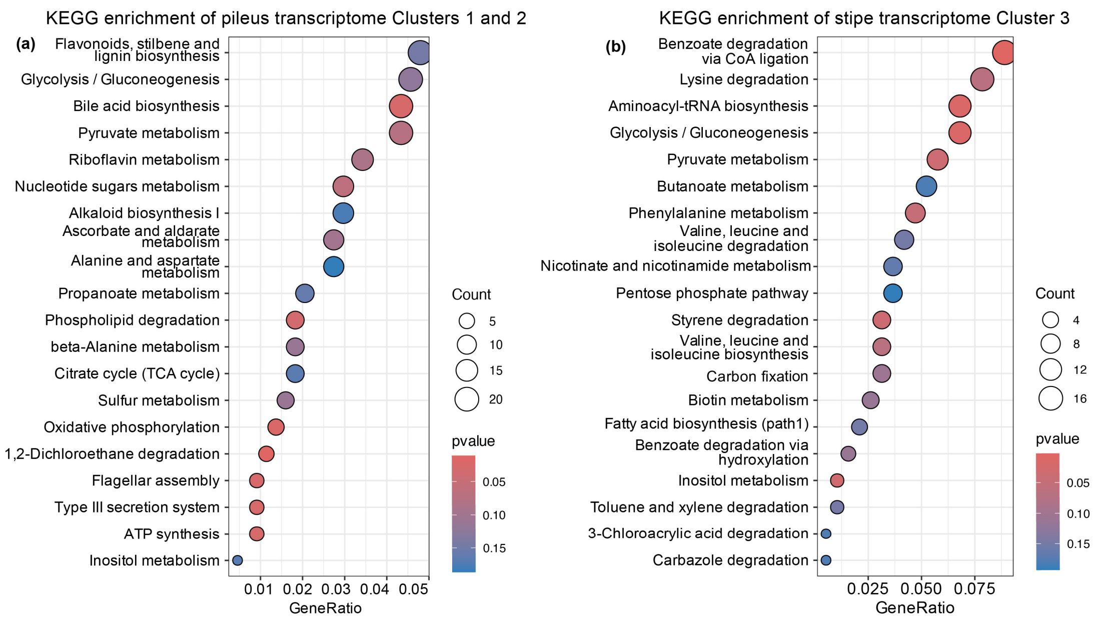

Supplementary Figure S4. KEGG enrichment analysis of selected transcriptome clusters in pileus and stipe tissues. (a) KEGG enrichment of pileus transcriptome Clusters 1 and 2. (b) KEGG enrichment of stipe transcriptome Cluster 3. The x-axis indicates the GeneRatio, bubble size represents the number of enriched genes, and color indicates the enrichment P value.

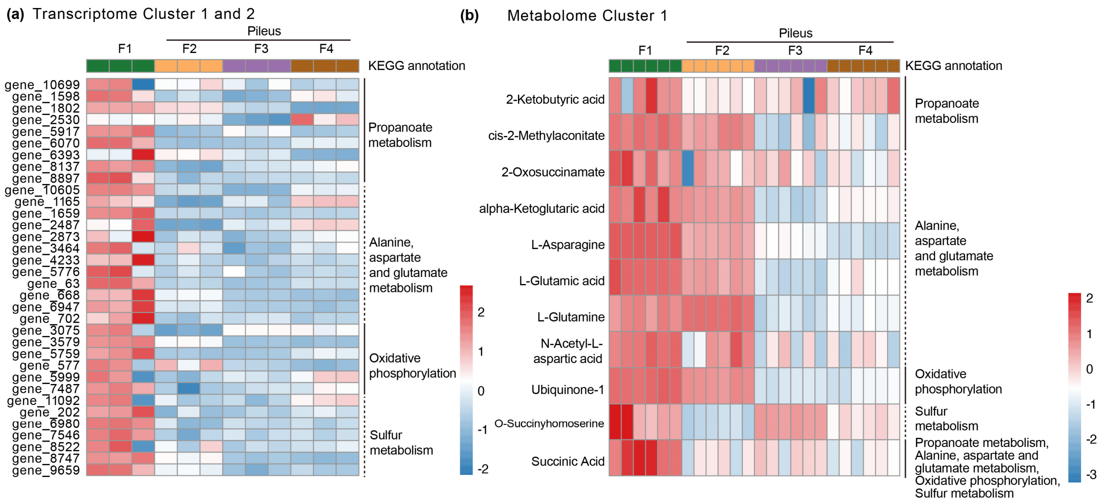

Supplementary Figure S5. Integrated transcriptomic and metabolomic analysis of shared KEGG pathways in pileus tissue. Heatmaps show the expression patterns of genes from pileus transcriptome Clusters 1 and 2 (a) and the abundance patterns of metabolites from Metabolome Cluster 1 (b) that were annotated to the same KEGG pathways. Samples are arranged according to developmental stage (F1-F4), and the shared KEGG pathways are indicated on the right. Color intensity represents relative gene expression or metabolite abundance.

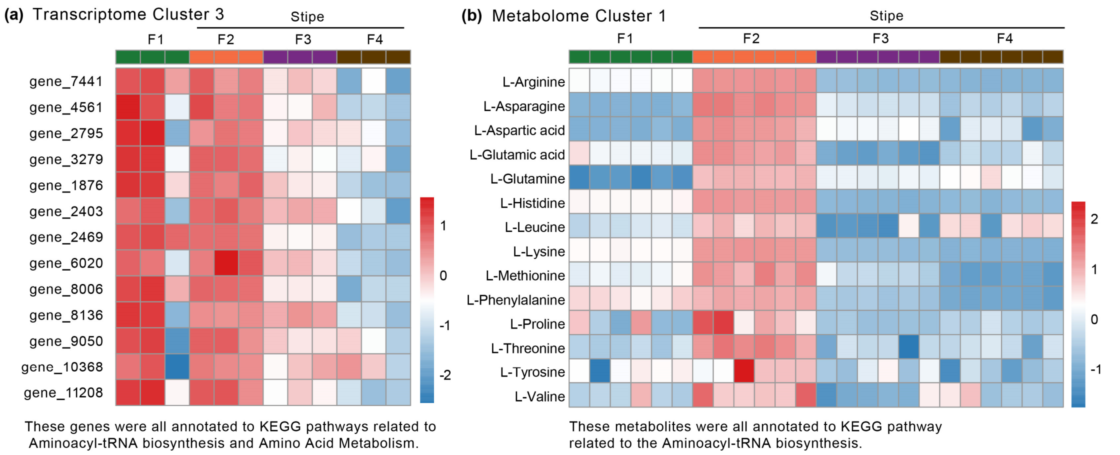

Supplementary Figure S6. Integrated transcriptomic and metabolomic analysis of shared KEGG pathways in stipe tissue. Heatmaps show the expression patterns of genes from stipe transcriptome Cluster 3 (a) and the abundance patterns of metabolites from Metabolome Cluster 1 (b) that were annotated to the same KEGG pathways related to aminoacyl-tRNA biosynthesis and amino acid metabolism. Samples are arranged according to developmental stage (F1–F4), and color intensity represents relative gene expression or metabolite abundance.
